# Characterization of the complete chloroplast genome of *Salvia leucantha* (Lamiaceae)

**DOI:** 10.1080/23802359.2021.2000899

**Published:** 2021-11-12

**Authors:** Yan Zhou, Hongrui Zhang, Hongming Ping, Yining Ding, Saiwen Hu, Guangyao Bi, Cuicui Li, Heming Li, Yong Huang, Linxiu Guo, Xingli Ma, Zhi Xia

**Affiliations:** a College of Agronomy, Henan Agricultural University, Zhengzhou, China; b School of Computer Science, University of Nottingham Ningbo China, Ningbo, China

**Keywords:** *Salvia leucantha*, *Salvia* subgenus *Calosphace*, chloroplast genome, Lamiaceae

## Abstract

*Salvia leucantha* (Lamiaceae) is an important horticultural plant with great ornamental and economic value. Here, we report the complete chloroplast genome of this species. The chloroplast genome was determined to be 151021 bp and the GC contents was 38.0%. The sequence includes a large single copy (LSC) region of 82,262 bp, a small single copy (SSC) region of 17,537 bp, and two separated inverted regions of 25,611 bp each. It contains 130 unique genes, including 85 protein-coding genes, 37 tRNA genes, and 8 rRNA genes. Based on the chloroplast genomes data of 26 species in *Salvia*, our result indicated that *S. leucantha*, *S. tiliifolia*, *S. hispanica,* and *S. splendens* formed one clade with Bootstrap = 100%. The four species belong to *Salvia* subgenus *Calosphace*, and *S. leucantha* was closely related to *Salvia tiliifolia* and *Salvia hispanica*. This result will facilitate population, genetic diversity and phylogenetic studies of *S. leucantha*.

*Salvia leucantha* Cav. (common name Mexican bush sage) is a horticultural species belonging to the family Lamiaceae and originally distributed in Mexico (González-Gallegos et al. 2020). As one of introduced species, it is widely cultivated in China for the beauty of its long inflorescences. Neoclerodane diterpenoids and related compounds as major components have been isolated from the aerial parts of *S. leucantha* of the subgenus Calosphace (Rojas et al. 2010). In recent years, the complete chloroplast genome of some species in *Salvia* were reported (Zhang et al. 2020; Zhao et al. 2020). However, the complete chloroplast genome of *Salvia leucantha* was not reported. In this study, we first reported the complete chloroplast genome of *S. leucantha*, and inferred phylogenetic relationships of this species to other taxa of genus *Salvia* in Lamiaceae.

The fresh leaves of *S. leucantha* were collected in Zhongmou country, Henan Province, China (34°46′55″N, 114°0′20″E). A specimen was deposited at Herbarium of Henan Agricultural University (HEAC) (contact person and email: Zhi Xia, xiazhiemail@126.com) under the voucher number XZ-2021-01. The total genomic DNA was extracted following the CTAB method (Doyle and Doyle 1987). After DNA extraction, 1 μg genomic DNA was randomly fragmented by Covaris, followed by fragment selection by Agencourt AMPure XP-Medium kit to an average size of 200–400 bp. Then the genomic library (paired-end, PE 150 bp) was sequenced on BGISEQ-500 platform at Beijing Genomics Institute (Shenzhen, China). Totally, 2.12 Gb sequence reads were obtained and used to assemble the chloroplast genome de novo by using NOVOPlasty (Dierckxsens et al. 2017) after trimming adaptor sequences and filtering low-quality reads. Then the assembled genome was annotated by using Geneious version 11.0.3 (Kearse et al. 2012) with manual adjustment. *Salvia hispanica* L. (GenBank accession number: MT083896) was used as reference plastid genome for assembling and annotation (Zhang et al. 2020). The annotated GenBank files of complete chloroplast sequence were submitted to GeneBank with the accession number MZ325864.

The complete chloroplast genome of *S. leucantha* has a length of 151,021 bp with a typical circular structure and a GC content of 38.0%. It consists of a large single copy region (LSC, 82,262 bp, 36.2% GC content), a small single-copy region (SSC, 17,537 bp, 31.7% GC content), and two inverted repeat regions (IR, 25,611 bp, 43.1% GC content). The genome harbors 130 genes, including 85 protein-coding genes, 37 tRNA genes, and 8 rRNA genes. Among these, 15 genes (*trn*K-UUU, *rps*16, *trn*G-UCC, *atp*F, *rpo*C1, *trn*L-UAA, *trn*V-UAC, *pet*B, *pet*D, *rpl*16, *rpl*2, *ndh*B, *trn*I-GUA, *trn*A-UGC, and *ndh*A) had one intron, while three genes (*rps*12, *clp*P, and *ycf*3) contained two introns.

A total of 25 complete chloroplast genomes in *Salvia* (Lamiaceae) together with the chloroplast genomes of *S. leucantha* in this study were utilized to clarify the phylogenetic position of *S. leucantha*. Based on the result of Zhao et al. (2020), Li et al. (2016) and Drew et al, (2017), we selected the *Melissa officinalis* L. and *Melissa yunnanensisas* C. Y. Wu et Y. C. Huang as outgroup. The sequence alignment was conducted with MAFFT v7.3 (Katoh and Standley 2013). A maximum likelihood analysis was performed with the RA × ML software (Stamatakis 2014) using 1000 bootstrap replicates. These analyses used the GTR substitution model with gamma-distributed rate heterogeneity among sites and the proportion of invariable sites estimated from the data. The phylogenetic tree indicates that all sampled species in *Salvia* formed a monophyletic group [Bootstrap (BS)=100%)] (Figure 1). Our results are in line with the most recent phylogeny of the group (Fragoso-Martínez et al. 2018). Within the genus *Salvia*, *S. leucantha*, *Salvia tiliifolia* Vahl, *S. hispanica* and *Salvia splendens* Sellow ex Wied-Neuw formed a clade with Bootstrap = 100%. The four species were relatively closely related in Fragoso-Martínez et al. (2018) phylogeny and they all were included in the most recently derived clade: core Calosphace. It can be clarified that *S. leucantha* being close to *S. tiliifolia* and *S. hispanica*. The chloroplast genomes resource may be utilized for DNA barcoding, conservation genetics, and breeding of *S. leucantha* in the future.

**Figure 1.**
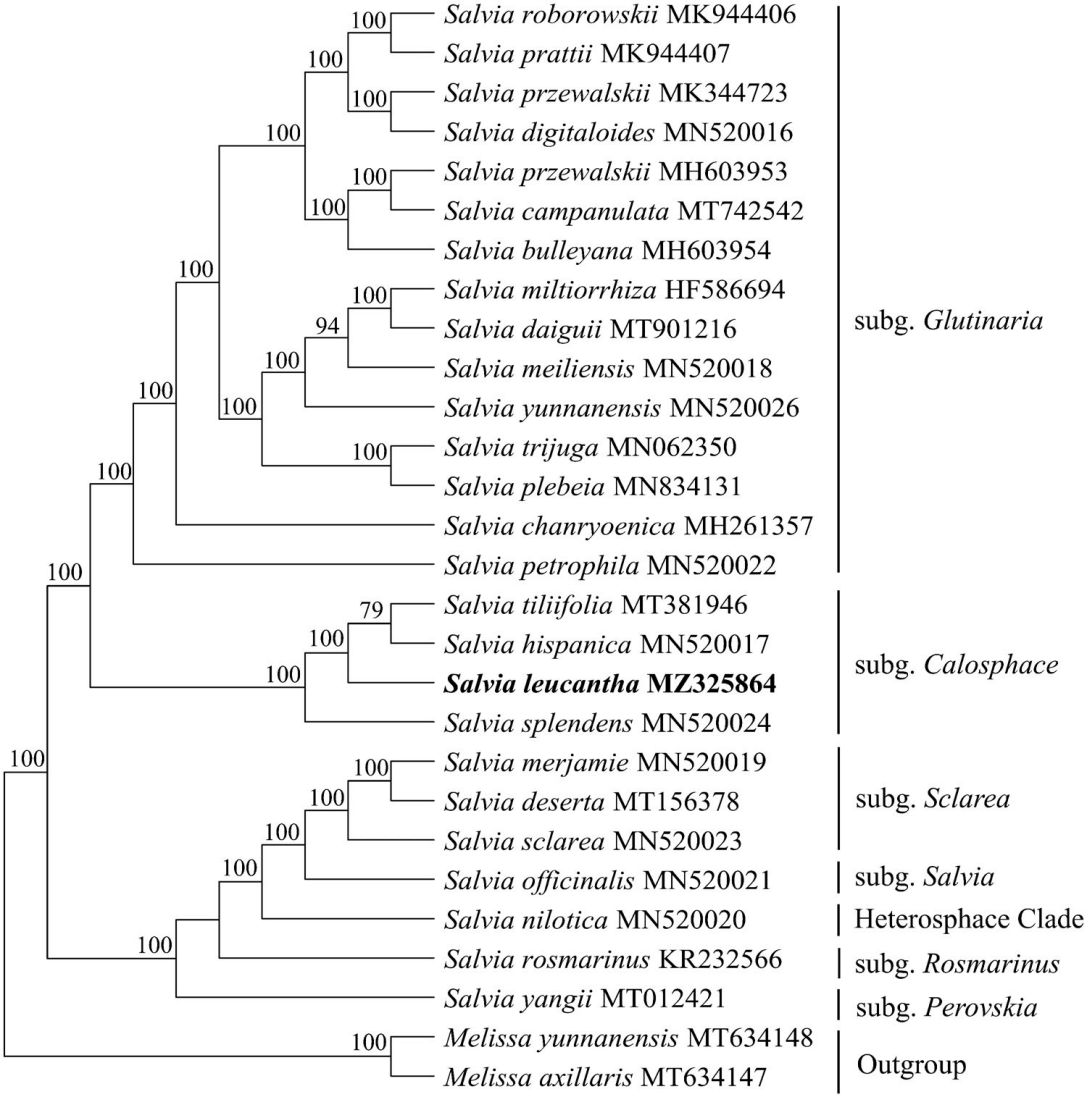
Maximum likelihood phylogenetic tree based on 26 complete chloroplast genome sequences of Salvia. The number on each node indicates the bootstrap value ▪.

## Data Availability

The complete chloroplast genome sequence data that support the findings of this study are openly available in GenBank of NCBI at (https://www.ncbi.nlm.nih.gov/) under the accession no. MZ325864. The associated BioProject, SRA, and Bio-Sample numbers are PRJNA746149, SRR15115656, and SAMN20192381, respectively.
